# Hospitalization deficit of in‐ and outpatient cases with cardiovascular diseases and utilization of cardiological interventions during the COVID‐19 pandemic: Insights from the German‐wide helios hospital network

**DOI:** 10.1002/clc.23549

**Published:** 2021-01-26

**Authors:** Sebastian König, Laura Ueberham, Vincent Pellissier, Sven Hohenstein, Andreas Meier‐Hellmann, Holger Thiele, Vusal Ahmadli, Michael A. Borger, Ralf Kuhlen, Gerhard Hindricks, Andreas Bollmann, Helios hospitals, Germany

**Affiliations:** ^1^ Heart Center Leipzig at University of Leipzig Department of Electrophysiology Leipzig Germany; ^2^ Leipzig Heart Institute Leipzig Germany; ^3^ Helios Hospitals Berlin Germany; ^4^ Heart Center Leipzig at University of Leipzig Department of Cardiology Leipzig Germany; ^5^ Heart Center Leipzig at University of Leipzig Department of Cardiac Surgery Leipzig Germany; ^6^ Helios Health Berlin Germany

**Keywords:** cardiovascular hospitalizations, cardiovascular procedures, COVID‐19, SARS‐CoV‐2

## Abstract

**Background:**

Treatment numbers of various cardiovascular diseases were reduced throughout the early phase of the ongoing COVID‐19 pandemic. Aim of this study was to (a) expand previous study periods to examine the long‐term course of hospital admission numbers, (b) provide data for in‐ and outpatient care pathways, and (c) illustrate changes of numbers of cardiovascular procedures.

**Methods and Results:**

Administrative data of patients with ICD‐10‐encoded primary diagnoses of cardiovascular diseases (heart failure, cardiac arrhythmias, ischemic heart disease, valvular heart disease, hypertension, peripheral vascular disease) and in‐ or outpatient treatment between March, 13th 2020 and September, 10th 2020 were analyzed and compared with 2019 data. Numbers of cardiovascular procedures were calculated using OPS‐codes. The cumulative hospital admission deficit (CumAD) was computed as the difference between expected and observed admissions for every week in 2020. In total, 80 hospitals contributed 294 361 patient cases to the database without relevant differences in baseline characteristics between the studied periods. There was a CumAD of −10% to −16% at the end of the study interval in 2020 for all disease groups driven to varying degrees by both reductions of in‐ and outpatient case numbers. The number of performed interventions was significantly reduced for all examined procedures (catheter ablations: −10%; cardiac electronic device implantations: −7%; percutaneous cardiovascular interventions: −9%; cardiovascular surgery: −15%).

**Conclusions:**

This study provides data on the long‐term development of cardiovascular patient care during the COVID‐19 pandemic demonstrating a significant CumAD for several cardiovascular diseases and a concomitant performance deficit of cardiovascular interventions.

## 1 INTRODUCTION

During the course of the COVID‐19 pandemic, reduced hospitalization rates were described for multiple acute cardiovascular and non‐cardiovascular diseases.^1^, ^2^, ^3^, ^4^, ^5^, ^6^, ^7^, ^8^, ^9^, ^10^, ^11^, ^12^, ^13^, ^14^ Since most of the previous investigations focused on the early phase of the pandemic in spring 2020, data concerning the development of hospitalization rates following April 2020 are scarce.^15^, ^16^, ^17^, ^18^ Evidence of an increased case‐severity respective mortality, especially in patients with cardiovascular diseases, led to concern that those reduced treatment numbers could negatively affect patients' long‐term outcome.^9^, ^15^, ^19^ Therefore, understanding patients' care pathways during the ongoing pandemic is of huge interest. Our group already introduced the cumulative hospitalization deficit as a metric to monitor cardiovascular hospitalizations across a multicenter hospital network in Germany.^20^ This study expands these data with regard to the examined study period, provides data also on outpatient treatment and highlights the numbers of different cardiological interventions.

## 2 METHODS

### 2.1 Data collection

Administrative data of 86 Helios hospitals in Germany were analyzed. Consecutive patient cases with an in‐ or outpatient hospital admission date between March 13th 2020 (start of the national protection phase according to the national pandemic plan for Germany) and September 10th 2020 were analyzed and compared with the corresponding period in 2019 (March 15th to September 12th 2019, changing dates relate to shifted week days). Cause‐specific hospitalizations were defined based on the encoded primary diagnosis at hospital discharge according to the International Statistical Classification of Diseases and Related Health Problems (ICD‐10‐GM [German Modification]). Patients with primary discharge diagnosis of specific cardiovascular diseases were further studied. Numbers of cardiovascular interventions were calculated using the Operations and Procedures‐codes (OPS [German adaptation of the International Classification of the Procedures in Medicine of the World Health Organization, version 2017]) within hospital discharge data. Comorbidities were identified from encoded secondary diagnoses at hospital discharge according to the Charlson Comorbidity Index (CCI), which has been calculated with minor adjustments according to previous publications.^21^, ^22^, ^23^ Patients in whom a laboratory‐proven infection with SARS‐CoV‐2 was encoded (ICD‐10 code: U07.1) were excluded. Detailed information about used ICD‐ and OPS‐codes as well as the participating hospitals is provided in the (Supplemental Tables 1–3). According to the data from the Robert‐Koch‐Institute, the German government's central scientific institution in the field of biomedicine, numbers of COVID‐19 cases per 100 000 inhabitants within a federal state were calculated and tertiles were computed to define the variable COVID‐19 case volume with corresponding low (<185.8), intermediate (185.8–404.0), and high (>404.0) COVID‐19 case volume.^24^ Hospitals were categorized with respect to the number of hospital admissions or procedures performed per center during the control period (2019) and further expressed as tertiles (hospital admissions: low <612 admissions/2019; intermediate 612–1970 admissions/2019; high >1970 admissions/2019; procedures: low <220 procedures/2019; intermediate 220–902 procedures/2019; high >902 procedures/2019). Information on in‐ or outpatient treatment was gathered from hospital discharge data. Patients' data were stored in a double‐pseudonymized form and data use was approved by the Helios Kliniken GmbH data protection authority. Considering the retrospective analysis of double‐pseudonymized administrative clinical routine data, ethics committee approval was determined not to be required in accordance with German law (professional regulation of Saxony §15) and informed consent was not obtained.

### 2.2 Statistical analysis

Administrative data were extracted from QlikView (QlikTech, Radnor, PA). We calculated the total number of monthly and weekly admissions, with weeks defined so that the first day of the national protection phase (Friday, March 13th 2020) corresponds to the beginning of the investigated week. The cumulative hospital admission deficit (CumAD) was computed as the difference between the expected and observed cumulative admission number for every week in 2020, expressed as a percentage (95% confidence interval [CI]) of the cumulative expected number, which is defined as the weekly average across the time interval in 2019. The difference between the expected and observed cumulative admission number was assessed using a χ^2^ test for the last week of the period. The p‐values were adjusted for multiple comparisons using a Bonferroni correction. For all tests we apply a two‐tailed 5% error criterion for significance.

## 3 RESULTS

### 3.1 Study cohort

A total of 80 hospitals contributed 294 361 patient cases to the database (6 hospitals did not treat cardiovascular patients meeting the inclusion criteria) for the years 2019 and 2020 (140 658 in total and 92 082 during the study period in 2020; 153 703 in total and 106 544 during the control period in 2019). There were no differences in the distribution of baseline characteristics between the control period in 2019 and each of the studied months in 2020 with respect to gender, age groups, CCI or the treatment in areas with different COVID‐19 case volumes (Table 1).

**TABLE 1:** Baseline characteristics of the total cohort (in‐ and outpatient) as a comparison of the average of 2019 with 2020 on a monthly basis

Variable	Monthly average 2019	January 2020	February 2020	March 2020	April 2020	May 2020	June 2020	July 2020	August 2020	September 2020	p
Total admissions	18 512	21 967	19 275	15 942	12 379	15 757	17 561	17 489	15 514	5784	
**Sex**											
Male	10 391 (56%)	12 313 (56%)	10 780 (56%)	9145 (57%)	7026 (57%)	8951 (57%)	9977 (57%)	9713 (56%)	8881 (57%)	3297 (57%)	n.s.
Female	8120 (44%)	9654 (44%)	8495 (44%)	6797 (43%)	5353 (43%)	6806 (43%)	7584 (43%)	7776 (44%)	6633 (43%)	2487 (43%)	n.s.
**Age group**											
≤64 years	6118 (33%)	7178 (33%)	6289 (33%)	5617 (35%)	4212 (34%)	5221 (33%)	5647 (32%)	5527 (32%)	4943 (32%)	1861 (32%)	n.s.
65–74 years	4274 (23%)	5166 (24%)	4448 (23%)	3670 (23%)	2854 (23%)	3738 (24%)	4185 (24%)	4134 (24%)	3699 (24%)	1401 (24%)	n.s.
≥ 75 years	8119 (44%)	9623 (44%)	8538 (44%)	6655 (42%)	5313 (43%)	6798 (43%)	7729 (44%)	7828 (45%)	6872 (44%)	2522 (44%)	n.s.
**Charlson comorbidity index**											
0–1	10 852 (59%)	13 311 (61%)	11 655 (60%)	9642 (60%)	7105 (57%)	9242 (59%)	10 351 (59%)	10 296 (59%)	9052 (58%)	3508 (61%)	n.s.
2–4	5661 (31%)	6399 (29%)	5679 (29%)	4630 (29%)	3828 (31%)	4789 (30%)	5333 (30%)	5338 (31%)	4819 (31%)	1739 (30%)	n.s.
≥5	1998 (11%)	2257 (10%)	1941 (10%)	1670 (10%)	1446 (12%)	1726 (11%)	1877 (11%)	1855 (11%)	1643 (11%)	537 (9%)	n.s.
**Admission type** ^a^											
Regular	6288 (34%)	6961 (32%)	6138 (32%)	5021 (31%)	3244 (26%)	4588 (29%)	5695 (32%)	5549 (32%)	4856 (31%)	2006 (35%)	n.s.
Urgent	7225 (39%)	8359 (38%)	7400 (38%)	6239 (39%)	5516 (45%)	6524 (41%)	6692 (38%)	6742 (39%)	6278 (40%)	2190 (38%)	n.s.
**Hospital volume**											
High	13 457 (73%)	15 889 (72%)	13 939 (72%)	11 550 (72%)	9131 (74%)	11 588 (74%)	13 031 (74%)	12 937 (74%)	11 463 (74%)	4333 (75%)	n.s.
Intermediate	4218 (23%)	5105 (23%)	4543 (24%)	3771 (24%)	2807 (23%)	3605 (23%)	3924 (22%)	3921 (22%)	3534 (23%)	1289 (22%)	n.s.
Low	836 (5%)	973 (4%)	793 (4%)	621 (4%)	441 (4%)	564 (4%)	606 (3%)	631 (4%)	517 (3%)	162 (3%)	n.s.
**COVID‐19 case volume**											
Low	7161 (39%)	8575 (39%)	7417 (38%)	6270 (39%)	4986 (40%)	6435 (41%)	6951 (40%)	6863 (39%)	6090 (39%)	2348 (41%)	n.s.
Intermediate	7137 (39%)	8639 (39%)	7715 (40%)	6289 (39%)	4753 (38%)	6045 (38%)	6860 (39%)	6645 (38%)	5932 (38%)	2143 (37%)	n.s.
High	4213 (23%)	4753 (22%)	4143 (21%)	3383 (21%)	2640 (21%)	3277 (21%)	3750 (21%)	3981 (23%)	3492 (23%)	1293 (22%)	n.s.

^a^ As some admissions are not classified, the total does not add to 100%.

### 3.2 Hospital admissions

**FIGURE 1:**
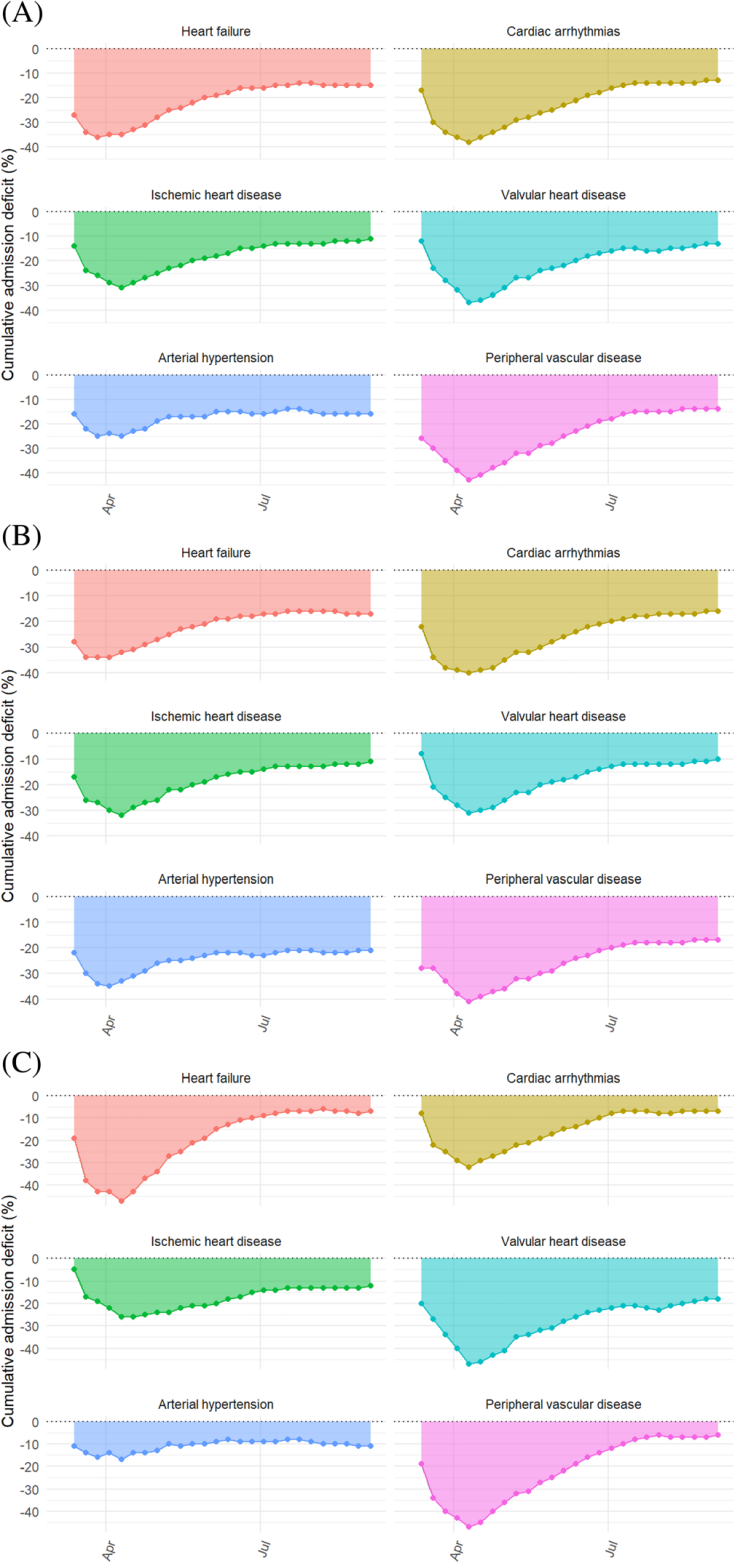
Total cumulative hospital admission deficit for different cardiovascular diseases in 2019 compared to 2020. A: cumulative admission deficit (total), B: cumulative admission deficit (inpatient cases), C: cumulative admission deficit (outpatient cases)

**FIGURE 2:**
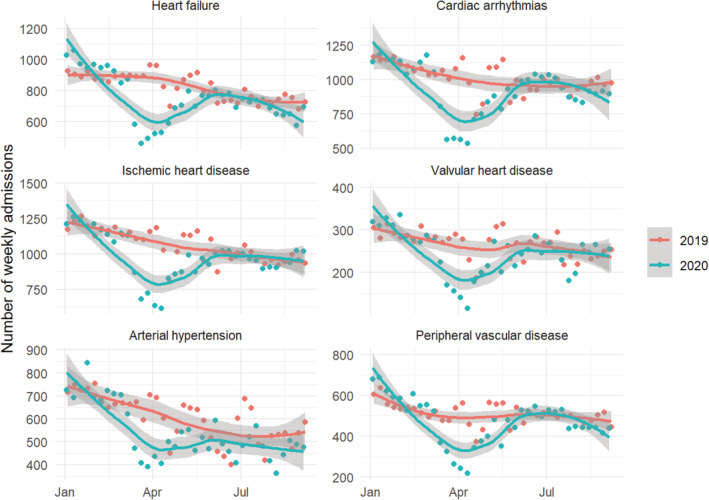
Comparison of weekly admission numbers between 2019 and 2020 per disease group

There was a significant CumAD of −10% to −16% at the end of the study period for all investigated cardiovascular disease groups. In detail, the overall CumAD was −15% (95%CI: −16; −14; p<0.001) for heart failure, −13% for cardiac arrhythmias (95%CI: −14; −12; p<0.001), −11% for ischemic heart disease (95%CI: −12; −10; p<0.001); −13% for valvular heart disease (95%CI: −15; −11; p<0.001), −16% for arterial hypertension‐related hospital admissions (95%CI: −17; −15; p<0.001), and −14% for admissions of patients with peripheral vascular disease (95%CI: −15; −13; p<0.001). The total reduction in hospital admissions was driven in all disease groups by a decline in both in‐ and outpatient treatment numbers with a greater reduction in inpatient cases in heart failure (inpatient −17% vs. outpatient −7%), cardiac arrhythmias (inpatient −16% vs. outpatient −7%), arterial hypertension (inpatient −21% vs. outpatient −11%), and peripheral vascular disease (inpatient −17% vs. outpatient −6%), a similar reduction of in‐ and outpatient treatment numbers in the group of ischemic heart disease (inpatient −11% vs. outpatient −12%) and a pronounced reduction of outpatient cases in valvular heart disease (inpatient −10% vs. outpatient −18%). All results of the CumAD analysis are shown in Table 2. When examining the time course of those effects there was a distinct decline in the CumAD in March and April with a disease‐specific recovery phase thereafter, although an overall deficit remained until the end of the observational period (Figure 1). Weekly admission rates per disease group were computed showing lower admission rates in 2020 with diverging curves from mid‐February (heart failure, cardiac arrhythmias, ischemic heart disease) or early March (valvular heart disease, arterial hypertension, peripheral vascular disease) until mid‐ to late‐May (except from arterial hypertension with early May). Results are illustrated in Figure 2.

**TABLE 2:** Cumulative hospital admission deficit for all subgroups of cardiovascular diseases and cardiovascular procedures

Admission until the final week (4 September‐10 September)				
	Expected	Observed	Hospitalization deficit (95% CI)	p
**Heart failure**				
Inpatients	17 232	14 321	−17% (−18; −16)	<0.001
Outpatients	3447	3189	−7% (−10; −5)	<0.001
Total	20 679	17 510	−15% (−16; −14)	<0.001
**Cardiac arrhythmias**				
Inpatients	16 971	14 286	−16% (−17; −15)	<0.001
Outpatients	8346	7721	−7% (−9; −6)	<0.001
Total	25 317	22 007	−13% (−14; −12)	<0.001
**Ischemic heart disease**				
Inpatients	20 609	18 338	−11% (−12; −10)	<0.001
Outpatients	5780	5063	−12% (−14; −11)	<0.001
Total	26 389	23 401	−11% (−12; −10)	<0.001
**Valvular heart disease**				
Inpatients	4277	3841	−10% (−12; −8)	<0.001
Outpatients	2379	1952	−18% (−21; −15)	<0.001
Total	6656	5793	−13% (−15; −11)	<0.001
**Arterial hypertension**				
Inpatients	7095	5626	−21% (−22; −19)	<0.001
Outpatients	7546	6707	−11% (−13; −9)	<0.001
Total	14 641	12 333	−16% (−17; −15)	<0.001
**Peripheral vascular disease**				
Inpatients	9120	7536	−17% (−19; −16)	<0.001
Outpatients	3742	3502	−6% (−9; −4)	<0.001
Total	12 862	11 038	−14% (−15; −13)	<0.001
**Catheter ablations**				
Total	4481	4039	−10% (−12; −8)	<0.001
**CIED implants**				
Total	5826	5391	−7% (−9; −6)	<0.001
**Cardiovascular surgery**				
Total	17 375	14 819	−15% (−16; −14)	<0.001
**Percutaneous cardiovascular interventions**				
Total	18 469	16 774	−9% (−10; −8)	<0.001

Abbreviation: CIED: cardiac implantable electronic device.

### 3.3 Cardiovascular interventions

**FIGURE 3:**
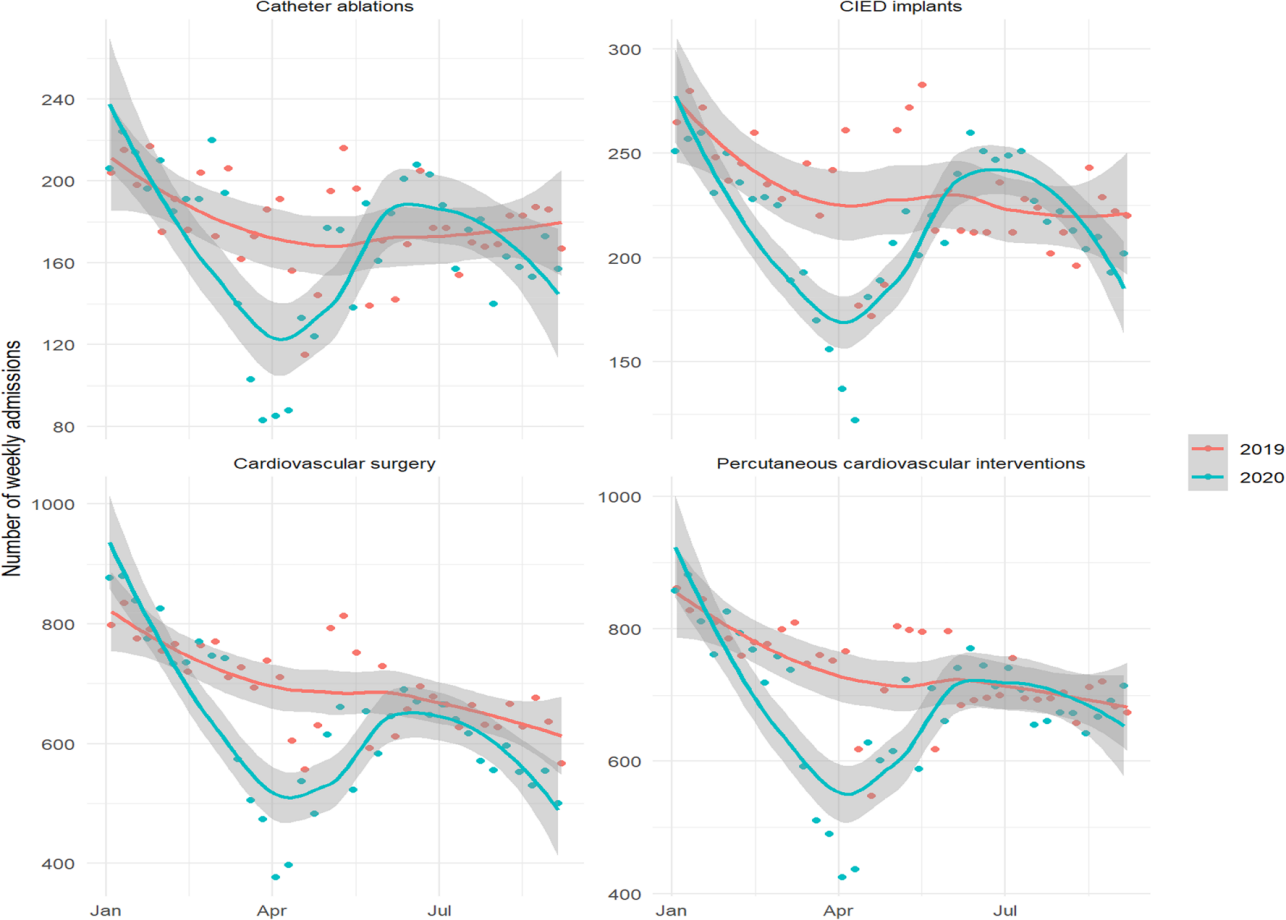
Comparison of weekly admission numbers between 2019 and 2020 per cardiovascular procedure

In total, 117 782 interventions were counted (56 193 in total and 37 620 during the study period in 2020; 61 589 in total and 42 638 during the control period in 2019). Baseline characteristics were not different in the subgroup of patients who underwent any prespecified cardiovascular procedure comparing 2019 and each of the studied months in 2020 (Supplemental Table 4). The cumulative number of performed interventions was significantly reduced for all examined procedures with −10% for catheter ablations (95%CI: −12; −8; p<0.001), −7% for cardiac implantable electronic device (CIED) operations (95%CI: −9; −6; p<0.001), −9% for percutaneous cardiovascular interventions (95%CI: −10; −8; p<0.001) and even −15% for cardiovascular surgery (95%CI: −16; −14; p<0.001). There has been no distinction made between in‐ and outpatient cases. Comparing weekly performance rates for each procedure group, a declining number of interventions was apparent from early (CIED implants) or mid‐February (catheter ablations, cardiovascular surgery, percutaneous coronary interventions) to early or mid‐June with a slight overcompensation period from end‐June to early August within catheter ablations and CIED implants without reaching statistical significance for this temporal increase (Figure 3).

## 4 DISCUSSION

The COVID‐19 pandemic already led to profound restructuring processes in the affected health care systems. We investigated an administrative database with 294 361 in‐ and outpatient cases for temporal trend analysis in healthcare utilization related to several cardiovascular diseases. Extending the findings of a previous report of our working group, we showed a significant reduction of case numbers for all investigated cardiovascular diseases in the early phase of the pandemic from March to May with a recovery of admission rates to the end of the observational period in comparison to a previous year control period.^20^ However, a relevant CumAD remained both for in‐ and outpatient care pathways and a significant deficit of all investigated cardiovascular interventions was apparent with unknown implications for the future.

There are no comparable datasets examining both in‐ and outpatient case numbers during the pandemic course since most studies focused on inpatient treatment so far. The initial acute decline in hospitalization rates is in line with previous findings reported for patients with acute coronary syndromes, heart failure, and other acute cardiovascular conditions. Only scarce data are available for the other disease groups examined. Regarding peripheral vascular disease, there are small single‐center experiences pointing into the same direction of overall lower hospitalization numbers with consecutive more severe clinical courses.^25^ This is even more surprising being aware of the procoagulatory effects of SARS‐CoV‐2‐infection potentially inducing peripheral embolisms.^26^ As our analysis excluded patients with COVID‐19 disease, the latter effect should per se not influence our findings. Nevertheless, an unknown number of undiagnosed infections could still have a potential impact on the observations made. When looking for studies investigating hospitalization numbers of patients with cardiac arrhythmias, a nationwide cohort study from Denmark presented an overall reduction of 47% in hospital admissions with atrial fibrillation.^27^ This is in line with our findings almost reaching a deficit of 40% for the group of all cardiac arrhythmias in April 2020, which corresponds to the observational period of Holt and colleagues.

The reduced performance rate of percutaneous coronary interventions is most likely to be explained by both the reduction of admission rates for acute coronary syndromes as described previously and the postponement of elective coronary interventions during the initial phase of the pandemic from March to early May as recommended by cardiological societies.^28^ Our data did not provide information on the interventional treatment within specific disease groups and could therefore not show whether patients admitted with coronary artery diseases were treated equally compared to the pre‐pandemic phase. However, data from a Swedish registry indicates a similar interventional strategy in patients with acute coronary syndromes leading to the assumption that the pure reduction of admission numbers is responsible for our observations.^29^ A similar or even pronounced reduction of cardiac catheterizations and electrophysiological interventions was reported from a nationwide database of the UK.^30^ Studies on the number of catheter ablations during the COVID‐19 pandemic are missing for continental Europe, but recommendations from scientific societies to reduce planned procedures should also be the most important influencing factors here, especially because the majority of electrophysiological interventions are elective in nature.^28^, ^31^ An early report from Italy showed a significant reduction in urgent pacemaker implantation rates (−28%) compared with 2019 without differences in the composition of the cohort regarding age or presentation with total AV block or syncope.^13^ This again is in line with our observations within the early pandemic phase from March to April 2020. Of note, pacemaker implantations following aortic valve interventions were excluded in this analysis indicating a true reduction of emergency admission for severe bradycardia. Interestingly, even in the field of acute aortic syndromes requiring surgery there were marked decreases in patient numbers reported from both Europe to the US.^32^, ^33^ Regardless of this, treatment numbers in cardiac surgery were reduced by ~50% up to 75% both in areas with low and even pronounced in those with high COVID‐19 case numbers.^34^ Once again, this is most likely due to a combination of the avoidance of patients entering the healthcare system or canceling their appointments, respectively, and healthcare providers postponing elective procedures according to the official recommendations.^35^, ^36^, ^37^, ^38^ Although there is no obvious explanation for the less pronounced effects shown in our data compared with some of the above mentioned studies, regional differences and the different COVID‐19 case numbers within the investigated cohorts must be taken into account in the interpretation.

Data regarding the development of admission rates following April 2020 are scarce and limited to two studies investigating acute coronary syndromes and one manuscript describing the changes in hospitalization rates for acute heart failure.^15^, ^16^, ^18^ Those three investigations showed a recovery phase in the later observational period similar to the increasing case numbers in the corresponding time interval seen in our analysis. However, comparability is limited as only patient cases up to May or mid‐June were included in previous works, which has now been extended to mid‐September in the present analysis.

There are several potential explanations for our observations both supporting the assumption of mere shifts in patients' care pathways and a potential true reduction of patient numbers. Firstly, patients' unwillingness of entering the healthcare system to avoid SARS‐CoV‐2‐exposition is one comprehensible reason for the reduction of hospital admissions. This is supported by an increased case‐severity both in patients with acute heart failure and acute myocardial infarctions indicating either patients' presentation at advanced disease stages or a selective admission of only the sickest patients. A prolonged time to first medical contact as well as to hospital admission in patients with acute coronary syndromes and equally reduced hospitalization rates both in areas with high and low COVID‐19 case volume are pointing in the same direction of an increased threshold for entering the healthcare system.^9^, ^39^ Moreover, the reduction of both in‐ and outpatient case numbers, which has now firstly been reported indicates such a connection. Secondly, the postponement of non‐urgent treatment in accordance to official recommendations will contribute to the observed findings. This is specifically of interest since Germany has one of the highest hospitalization rates for cardiovascular diseases across Europe.^40^ However, the majority of the above‐mentioned studies showing comparable hospitalization deficits only included medical emergencies, which should be less affected by the regular scheduling within the health care system. Furthermore, percentage changes in hospitalization numbers from other European countries or the US during spring are in a similar range not indicating that our observations are only caused to a previous overtreatment of cardiovascular patients specifically in Germany. Nevertheless, the persisting CumAD until the end of the observational period could potentially be influenced by the previously liberal handling of indications for inpatient care and corresponding procedures.

A misclassification of diseases in favor of COVID‐19 disease is possible for some disease groups with overlapping clinical presentation like heart failure. Nevertheless, as the decline of patient numbers was also observed in cohorts in which an overlap of symptoms is unlikely (peripheral vascular disease, acute stroke), this should only affect our findings to a minor extend. Factors being discussed to potentially truly reduce the necessity of in‐ our outpatient hospital treatment are an improved air quality during the lockdown phase and the reduction of seasonal infections as a consequence of restricted social contacts since both of them are triggers for an acute deterioration of pre‐existing heart diseases.^41^

Further studies are needed to confirm our findings and identify causes for the observations being made. Whether those changes in healthcare utilization during the COVID‐19 pandemic imply consequences for future treatment of patients with cardiovascular diseases cannot yet be foreseen.

## 5 LIMITATIONS

This study analyzed administrative data, which were not stored for research interests, but for remuneration reasons, which potentially could affect the encoded information. Quality of the results depends to a large extent on the correct encoding of procedures and diagnoses at hospital discharge.^23^ This is particularly true for the encoding of SARS‐CoV‐2‐infection, as the specific ICD‐code has been introduced at April 1st 2020 and was retrospectively encoded thereafter for all previous cases. Information regarding patients' specific medical history, cardiac imaging, laboratory results, medication, and treatment‐related data were not available due to the type and structure of the analyzed database. Moreover, the majority of outpatient care in Germany is provided by resident practitioners and therefore the present analysis only represents a selection of patients treated in the environment of a hospital. The comparison of admission numbers only with data from 2019 harbors the possibility that the observed effects are caused by year‐dependent fluctuations in admission numbers. However, a comparison with previous year's data have also been considered a valid method of comparison in several other studies investigating changes in health care utilization during the COVID‐19 pandemic.^1^, ^2^, ^3^, ^8^, ^9^, ^10^, ^11^, ^13^, ^14^, ^15^, ^27^, ^42^, ^43^, ^44^, ^45^

## 6 CONCLUSION

Our study is the first to show the development of hospital admission numbers during the course of the COVID‐19 pandemic for several cardiovascular disease groups demonstrating both a decrease in case numbers within the in‐ and outpatient setting. Although increasing after the early pandemic phase from March to May, a significant CumAD remained for all disease groups. Moreover, a significant performance deficit for all studied cardiovascular interventions was found. Consequences of these findings cannot be foreseen and deserve further research.

## CONFLICT OF INTEREST

Gerhard Hindricks is receiving grants through the Leipzig Heart Institute from Boston Scientific (Boston Scientific Corporation, Marlborough, MA), and Abbott/St. Jude Medical (Abbott Laboratories, Chicago, IL), no personal payments are to declare. All other authors state that there is nothing to declare.

**Abbreviations**
CCI: Charlson comorbidity index
CI: confidence interval
CumHD: cumulative hospital admission deficit
GLMM: generalized linear mixed models
ICD‐10‐GM: International Statistical Classification of Diseases and Related Health Problems (German Modification)
OPS: operations and procedures

## Supporting information


**Appendix** **S1. Supporting information**.

## Data Availability

The data underlying this article will be shared on reasonable request to the corresponding author.

## References

[1] Andersson C , Gerds T , Fosbol E , et al. Incidence of new‐onset and worsening heart failure before and after the COVID‐19 epidemic lockdown in Denmark: a Nationwide cohort study. Circ Heart Fail. 2020;35(10):3129‐3132. 10.1161/circheartfailure.120.007274. 32482087

[2] Baum A , Schwartz MD . Admissions to veterans affairs hospitals for emergency conditions during the COVID‐19 pandemic. JAMA. 2020;324(1):96‐99. 10.1001/jama.2020.9972. 32501493 PMC7275263

[3] Bhatt AS , Moscone A , McElrath EE , et al. Declines in hospitalizations for acute cardiovascular conditions during the COVID‐19 pandemic: a multicenter tertiary care experience. J Am Coll Cardiol. 2020;76(3):280‐288. 10.1016/j.jacc.2020.05.038. 32470516 PMC7250561

[4] Bollmann A , Hohenstein S , Meier‐Hellmann A , Kuhlen R , Hindricks G , Helios hospitals G . Emergency hospital admissions and interventional treatments for heart failure and cardiac arrhythmias in Germany during the Covid‐19 outbreak insights from the German‐wide Helios hospital network. Eur Heart J Qual Care Clin Outcomes. 2020;6(3):221‐222. 10.1093/ehjqcco/qcaa049. 32502261 PMC7314091

[5] Bromage DI , Cannata A , Rind IA , et al. The impact of COVID‐19 on heart failure hospitalization and management: report from a heart failure unit in London during the peak of the pandemic. Eur J Heart Fail. 2020;22:978‐984. 10.1002/ejhf.1925. 32478951 PMC7300902

[6] Clerici M , Durbano F , Spinogatti F , Vita A , de Girolamo G , Micciolo R . Psychiatric hospitalization rates in Italy before and during COVID‐19: did they change? An analysis of register data. Ir J Psychol Med. 2020;37(4):283‐290. 10.1017/ipm.2020.29. 32368994 PMC7264453

[7] Cox ZL , Lai P , Lindenfeld J . Deceases in acute heart failure hospitalizations during COVID‐19. Eur J Heart Fail. 2020;22:1045‐1046. 10.1002/ejhf.1921. 32469132 PMC7283634

[8] De Filippo O , D'Ascenzo F , Angelini F , et al. Reduced rate of hospital admissions for ACS during Covid‐19 outbreak in northern Italy. N Engl J Med. 2020;383(1):88‐89. 10.1056/NEJMc2009166. 32343497 PMC7224608

[9] De Rosa S , Spaccarotella C , Basso C , et al. Reduction of hospitalizations for myocardial infarction in Italy in the COVID‐19 era. Eur Heart J. 2020;41(22):2083‐2088. 10.1093/eurheartj/ehaa409. 32412631 PMC7239145

[10] Garcia S , Albaghdadi MS , Meraj PM , et al. Reduction in ST‐segment elevation cardiac catheterization laboratory activations in the United States during COVID‐19 pandemic. J Am Coll Cardiol. 2020;75(22):2871‐2872. 10.1016/j.jacc.2020.04.011. 32283124 PMC7151384

[11] Lau LH , Wong SH , Yip TC , Wong GL , Wong VW , Sung JJ . Collateral effect of COVID‐19 pandemic on hospitalizations and clinical outcomes in gastrointestinal and liver diseases ‐ a territory‐wide observational study in Hong Kong. Gastroenterology. 2020;159(5):1979‐1981. 10.1053/j.gastro.2020.07.042. 32721440 PMC7382332

[12] Metzler B , Siostrzonek P , Binder RK , Bauer A , Reinstadler SJ . Decline of acute coronary syndrome admissions in Austria since the outbreak of COVID‐19: the pandemic response causes cardiac collateral damage. Eur Heart J. 2020;41(19):1852‐1853. 10.1093/eurheartj/ehaa314. 32297932 PMC7184486

[13] Migliore F , Zorzi A , Gregori D , et al. Urgent pacemaker implantation rates in the Veneto region of Italy after the COVID‐19 outbreak. Circ Arrhythm Electrophysiol. 2020;13(6):e008722. 10.1161/CIRCEP.120.008722. 32434373 PMC7299094

[14] Oseran AS , Nash D , Kim C , et al. Changes in hospital admissions for urgent conditions during COVID‐19 pandemic. Am J Manag Care. 2020;26(8):327‐328. 10.37765/ajmc.2020.43837. 32835458

[15] Cannata A , Bromage DI , Rind IA , et al. Temporal trends in decompensated heart failure and outcomes during COVID‐19: a multisite report from heart failure referral centres in London. Eur J Heart Fail. 2020. 10.1002/ejhf.1986. PMC7461082 32809274

[16] Gluckman TJ , Wilson MA , Chiu ST , et al. Case rates, treatment approaches, and outcomes in acute myocardial infarction during the coronavirus disease 2019 pandemic. JAMA Cardiol. 2020;5 (12):1‐6. 10.1001/jamacardio.2020.3629. PMC7414426 32766756

[17] Konig S , Hohenstein S , Meier‐Hellmann A , et al. In‐hospital Care in Acute Heart Failure during the COVID‐19 pandemic: insights from the German‐wide Helios hospital network. Eur J Heart Fail. 2020. 10.1002/ejhf.2044. 33135851

[18] Mafham MM , Spata E , Goldacre R , et al. COVID‐19 pandemic and admission rates for and management of acute coronary syndromes in England. Lancet. 2020;396(10248):381‐389. 10.1016/S0140-6736(20)31356-8. 32679111 PMC7429983

[19] Colivicchi F , Di Fusco SA , Magnanti M , Cipriani M , Imperoli G . The impact of the coronavirus Disease‐2019 pandemic and Italian lockdown measures on clinical presentation and Management of Acute Heart Failure. J Card Fail. 2020;26(6):464‐465. 10.1016/j.cardfail.2020.05.007. 32417376 PMC7224656

[20] Bollmann A , Pellissier V , Hohenstein S , et al. Cumulative hospitalization deficit for cardiovascular disorders in Germany during the Covid‐19 pandemic. Eur Heart J Qual Care Clin Outcomes. 2020. 10.1093/ehjqcco/qcaa071. PMC7499594 32857835

[21] Konig S , Ueberham L , Muller‐Rothing R , et al. Catheter ablation of ventricular arrhythmias and in‐hospital mortality: insights from the German‐wide Helios hospital network of 5052 cases. Europace. 2020;22(1):100‐108. 10.1093/europace/euz260. 31638643

[22] Konig S , Ueberham L , Schuler E , et al. In‐hospital mortality of patients with atrial arrhythmias: insights from the German‐wide Helios hospital network of 161 502 patients and 34 025 arrhythmia‐related procedures. Eur Heart J. 2018;39(44):3947‐3957. 10.1093/eurheartj/ehy528. 30165430

[23] Quan H , Sundararajan V , Halfon P , et al. Coding algorithms for defining comorbidities in ICD‐9‐CM and ICD‐10 administrative data. Med Care. 2005;43(11):1130‐1139. 10.1097/01.mlr.0000182534.19832.83. 16224307

[24] Robert‐Koch‐Insitut. COVID‐19: case numbers in Germany Robert‐Koch‐Institut; last access 05th January 2021.

[25] Li W , Chen X , Feng H . Impact of COVID‐19 on peripheral arterial disease treatment. Ann Vasc Surg. 2020;67:6‐7. 10.1016/j.avsg.2020.05.045. 32502682 PMC7265821

[26] Mestres G , Puigmacia R , Blanco C , Yugueros X , Esturrica M , Riambau V . Risk of peripheral arterial thrombosis in COVID‐19. J Vasc Surg. 2020;72(2):756‐757. 10.1016/j.jvs.2020.04.477. 32417015 PMC7203033

[27] Holt A , Gislason GH , Schou M , et al. New‐onset atrial fibrillation: incidence, characteristics, and related events following a national COVID‐19 lockdown of 5.6 million people. Eur Heart J. 2020;41(32):3072‐3079. 10.1093/eurheartj/ehaa494. 32578859 PMC7337750

[28] (2020). The European Society of Cardiology. ESC Guidance for the Diagnosis and Management of CV Disease during the COVID‐19 Pandemic ESC Webpage.

[29] Mohammad MA , Koul S , Olivecrona GK , et al. Incidence and outcome of myocardial infarction treated with percutaneous coronary intervention during COVID‐19 pandemic. Heart. 2020;106:1812‐1818. 10.1136/heartjnl-2020-317685. 33023905 PMC7677488

[30] Mohamed MO , Banerjee A , Clarke S , et al. Impact of COVID‐19 on cardiac procedure activity in England and associated 30‐day mortality. Eur Heart J Qual Care Clin Outcomes. 2020. 10.1093/ehjqcco/qcaa079. PMC7665465 33079204

[31] Lakkireddy DR , Chung MK , Deering TF , et al. Guidance for rebooting electrophysiology through the COVID‐19 pandemic from the Heart Rhythm Society and the American Heart Association electrocardiography and arrhythmias Committee of the Council on clinical cardiology: endorsed by the American College of Cardiology. JACC Clin Electrophysiol. 2020;6(8):1053‐1066. 10.1016/j.jacep.2020.06.004. 32819525 PMC7291987

[32] Khot UN , Reimer AP , Brown A , et al. Impact of COVID‐19 pandemic on critical care transfers for ST‐segment‐elevation myocardial infarction, stroke, and aortic emergencies. Circ Cardiovasc Qual Outcomes. 2020;13(8):e006938. 10.1161/CIRCOUTCOMES.120.006938. 32524835

[33] Reyes Valdivia A , San Norberto E , Moreno R , et al. Massive drop in elective and urgent aortic procedures during the peak of the COVID‐19 outbreak in Spanish multicenter analysis. J Vasc Surg. 2020;73(1):349‐350. 10.1016/j.jvs.2020.08.027. 32890717 PMC7467013

[34] Gaudino M , Chikwe J , Hameed I , Robinson NB , Fremes SE , Ruel M . Response of cardiac surgery units to COVID‐19: an internationally‐based quantitative survey. Circulation. 2020;142(3):300‐302. 10.1161/CIRCULATIONAHA.120.047865. 32392425 PMC7365675

[35] Ad N , Luc JGY , Nguyen TC , Group C‐NACSSW . Cardiac surgery in North America and coronavirus disease 2019 (COVID‐19): regional variability in burden and impact. J Thorac Cardiovasc Surg. 2020;5223(6):31983‐31988. 10.1016/j.jtcvs.2020.06.077. PMC7330597 32768300

[36] Haft JW , Atluri P , Ailawadi G , et al. Adult cardiac surgery during the COVID‐19 pandemic: a tiered patient triage guidance statement. J Thorac Cardiovasc Surg. 2020;160(2):452‐455. 10.1016/j.jtcvs.2020.04.011. 32689701 PMC7161470

[37] Salenger R , Etchill EW , Ad N , et al. The surge after the surge: cardiac surgery post‐COVID‐19. Ann Thorac Surg. 2020;110(6):2020‐2025. 10.1016/j.athoracsur.2020.04.018. 32376350 PMC7196543

[38] Shah PB , Welt FGP , Mahmud E , et al. Triage considerations for patients referred for structural heart disease intervention during the COVID‐19 pandemic: an ACC/SCAI position statement. JACC Cardiovasc Interv. 2020;13(12):1484‐1488. 10.1016/j.jcin.2020.04.001. 32250751 PMC7270905

[39] Kwok CS , Gale CP , Kinnaird T , et al. Impact of COVID‐19 on percutaneous coronary intervention for ST‐elevation myocardial infarction. Heart. 2020. Epub 2020/09/02;106:1805‐1811. 10.1136/heartjnl-2020-317650. 32868280

[40] Eurostat . Hospital discharge rates for in‐patients with diseases of the circulatory system, 2018 Eurostat2020

[41] Pranata R , Vania R , Tondas AE , Setianto B , Santoso A . A time‐to‐event analysis on air pollutants with the risk of cardiovascular disease and mortality: a systematic review and meta‐analysis of 84 cohort studies. J Evid Based Med. 2020;13(2):102‐115.Epub 2020/03/14. 10.1111/jebm.12380. 32167232

[42] Antonucci M , Recupero SM , Marzio V , et al. The impact of COVID‐19 outbreak on urolithiasis emergency department admissions, hospitalizations and clinical management in Central Italy: a multicentric analysis. Actas Urol Esp. 2020;44(9):611‐616. 10.1016/j.acuro.2020.06.005.32713658PMC7332912

[43] Baldi E , Sechi GM , Mare C , et al. COVID‐19 kills at home: the close relationship between the epidemic and the increase of out‐of‐hospital cardiac arrests. Eur Heart J. 2020;41(32):3045‐3054. 10.1093/eurheartj/ehaa508. 32562486 PMC7337787

[44] Frankfurter C , Buchan TA , Kobulnik J , et al. Reduced rate of hospital presentations for heart failure during the COVID‐19 pandemic in Toronto, Canada. Can J Cardiol. 2020;36(10):1680‐1684. 10.1016/j.cjca.2020.07.006. 32682855 PMC7366087

[45] Mountantonakis SE , Saleh M , Coleman K , et al. Out‐of‐hospital cardiac arrest and acute coronary syndrome hospitalizations during the COVID‐19 surge. J Am Coll Cardiol. 2020;76(10):1271‐1273.Epub 2020/07/18. 10.1016/j.jacc.2020.07.021. 32679154 PMC7833304

