# Supplementary material for: Hospitalization deficit of in‐ and outpatient cases with cardiovascular diseases and utilization of cardiological interventions during the COVID‐19 pandemic: Insights from the German‐wide helios hospital network
Source: Clin Cardiol. 2021 Jan 26;44(3):392–400. doi: 10.1002/clc.23549 (PMC7943897; doi:10.1002/clc.23549)

**Hospitalization Deficit of In- and Outpatient Cases with Cardiovascular Diseases and Utilization of Cardiological Interventions during the COVID-19 Pandemic:**

**Insights from the German-wide Helios Hospital Network**

Sebastian König^1,2^, MD; Laura Ueberham^1,2^, MD; Vincent Pellissier^2^, PhD;

Sven Hohenstein^2^, PhD; Andreas Meier-Hellmann^3^, MD; Holger Thiele^2,4^, MD;

Vusal Ahmadli^2,4^, MD; Michael A. Borger^2,5^, MD; Ralf Kuhlen^6^, MD;

Gerhard Hindricks^1,2^, MD and Andreas Bollmann^1,2^, MD, PhD

on behalf of Helios hospitals, Germany

**Affiliations:**

1 - Heart Center Leipzig at University of Leipzig, Department of Electrophysiology, Leipzig, Germany

2 - Leipzig Heart Institute, Leipzig, Germany

3 - Helios Hospitals, Berlin, Germany

4 - Heart Center Leipzig at University of Leipzig, Department of Cardiology, Leipzig, Germany

5 - Heart Center Leipzig at University of Leipzig, Department of Cardiac Surgery, Leipzig, Germany

6 - Helios Health, Berlin, Germany

**Journal:** Clinical Research in Cardiology

**Corresponding author:**

Sebastian König

Heart Center Leipzig at University of Leipzig

Department of Electrophysiology

Strümpellstraße 39, 04289 Leipzig, Germany

Fax: +49-341-865-1460

E-mail: Sebastian.koenig@helios-gesundheit.de

ORCID-ID: 0000-0002-3759-3052

**Supplemental Material**

Supplemental Table 1: ICD-codes used as tracers for the identification of investigated cardiovascular disease groups, relevant comorbidities / clinical conditions and interventions

Supplemental Table 2: ICD-codes used to calculate Charlson Comorbidity Index

Supplemental Table 3: Participating centers

Supplemental Table 4: Baseline characteristics of the subgroup of cases with cardiovascular procedures as a comparison of the average of 2019 with 2020 on a monthly basis

Supplemental Figure 1: Graphical overview on case numbers of laboratory proven SARS-CoV-2 infections in Germany per day

Supplemental Table 1: ICD-codes used as tracers for the identification of investigated cardiovascular disease groups, relevant comorbidities / clinical conditions and interventions

| ICD*-COdes of underlying cardiovascular diseases, relevant comorbidities / clinical conditions and interventions | |
| --- | --- |
| Heart failure | I42.x; I43.x; I50.x |
| Cardiac arrhythmias | I44.x; I45.x; I47.x; I48.x; I49.x; R00.0; R00.1; R00.2 |
| Ischemic heart disease | I20.x; I21.x; I22.x; I23.x; I24.x; I25.x |
| Valvular heart disease | I05.x; I06.x; I07.x; I08.x; I34.x; I35.x; I36.x; I37.x; Q22.x; Q23.x; T82.0; Z95.2; Z95.3; Z95.4 |
| Arterial hypertension | I10.x; I11.x; I12.x; I13.x; I14.x; I15.x |
| Peripheral vascular disease | I70.x; I71.x; I72.x; I73.x; I74.x; I75.x; I76.x; I77.x; I78.x; I79.x |
| Obesity | E66 |
| SARS-CoV-2 infection, laboratory-proven | U07.1 |
| Catheter ablation | 8-835.x |
| CIED implants | 5-377.x; 5-378.x |
| Cardiovascular surgery | 5-35x; 5-36x; 5-37x (excluding: 5-377; 5-378); 5-38x; 5-39x |
| Percutaneous cardiovascular interventions | 8-836.x; 8-837.x; 8-838.x; 8-83d |

* International Statistical Classification of Diseases and Related Health Problems (ICD-10-GM [German Modification])

CIED: cardiac implantable electronic device

Supplemental Table 2: ICD-codes used to calculate Charlson Comorbidity Index

| ICD*-codes used to calculate CHarlson comorbidity index | | | |
| --- | --- | --- | --- |
| *Number* | *Condition* | *Weight* | *ICD*-10-GM-code* |
| 01 | Myocardial infarction | 1 | I21.x; I22.x; I25.2 |
| 02 | Congestive heart failure | 1 | I09.0; I11.0; I13.0; I13.2; I25.5; I42.0; I42.1; I42.2; I42.5-9; I43.x; I50.x |
| 03 | Peripheral vascular disease | 1 | I70.x; I71.x; I73.1; I73.8-9; I77.1; I79.0; I79.2; K55.1; K55.8-9; Z.95.8-9 |
| 04 | Cerebrovascular disease | 1 | I60.x; I61.x; I63.x; I64.x; I65.x; I66.x; I67.0; I67.2; I67.3; I67.8; I67.9; I69.x; G45.x; G46.x; H34.0 |
| 05 | Dementia | 1 | F00.x; F01.x; F02.x; F03.x; F05.1; G30.x; G31.1 |
| 06 | Chronic pulmonary disease | 1 | I27.8.; I27.9; J40.x; J41.x; J42.x; J43.x; J44.x; J45.x; J46.x; J47.x; J60.x; J61.x; J62.x; J63.x; J64.x; J65.x; J66.x; J67.x; J68.4; J70.1; J70.3 |
| 07 | Rheumatic disease | 1 | M05.x; M06.x; M31.5; M32.x; M33.x; M34.x; M35.1; M35.3; M36.0 |
| 08 | Peptic ulcer disease | 1 | K25.x; K26.x; K27.x; K28.x |
| 09 | Mild liver disease | 1 | B18.x; K70.0-3; K70.9; K71.3-5; K71.7; K73.x; K74.x; K76.0; K76.2-4; K76.8-9; Z94.4 |
| 10 | Diabetes without chronic complication | 1 | E10.0-1; E10.6; E10.8-9; E11.0-1; E11.6; E11.8-9; E12.0-1; E12.6; E12.8-9; E13.0-1; E13.6; E13.8-9; E14.0-1; E14.6; E14.8; E14.9 |
| 11 | Diabetes with chronic complication | 2 | E10.2-5; E10.7; E11.2-5; E11.7; E12.2-5; E12.7; E13.2-5; E13.7; E14.2-5; E14.7 |
| 12 | Hemiplegia or paraplegia | 2 | G04.1; G11.4; G80.1-2; G81.x; G82.x; G83.0-4; G83.9 |
| 13 | Chronic kidney disease | 2 | I12.0; I13.1; N03.2-7; N05.2-7; N18.x; N19.x; N25.0; Z49.0-2; Z94.0; Z99.2 |
| 14 | Any malignancy including lymphoma and leukemia except malignant neoplasm of skin | 2 | C00.x-C26.x; C30.x–C34.x; C37.x–C41.x; C43.x; C45.x-C58.x; C60.x-C76.x; C81.x-C85.x; C88.x; C90.x-C97.x |
| 15 | Moderate or severe liver disease | 3 | I85.0; I85.9; I86.4; I98.2; K70.4; K71.1; K72.1; K72.9; K76.5; K76.6; K76.7 |
| 16 | Metastatic solid tumor | 3 | C77.x; C78.x; C79.x; C80.x |
| 17 | AIDS / HIV | 6 | B20.x; B21.x; B22.x; B24.x |

* International Statistical Classification of Diseases and Related Health Problems (ICD-10-GM [German Modification])

**Supplemental Table 3: Participating centers**

| Participating centers | |
| --- | --- |
| *Center* | *Center* |
| Ambrock | Leipzig Parkkrankenhaus |
| Attendorn | Leisnig |
| Aue | Lengerich |
| Bad Berleburg | Lutherstadt Eisleben |
| Bad Gandersheim | Meiningen |
| Bad Kissingen | Müllheim |
| Bad Saarow | München Perlach |
| Bad Schwartau | München West |
| Berlin EVB | Niederberg |
| Berlin Buch | Nienburg |
| Blankenhain | Nordenham |
| Bleicherode | Northeim |
| Bochum | Oberhausen |
| Breisach | Oschersleben |
| Burg | Pforzheim |
| Cuxhaven | Pirna |
| Damp | Plauen |
| Dachau | Rottweil |
| Duisburg | Sahlenburg |
| Erfurt | Salzgitter |
| Erlenbach | Sangerhausen |
| Freital | Schkeuditz |
| Gifhorn | Schleswig |
| Gotha | Schwelm |
| Grebenhain | Schwerin |
| Hamburg, ENDO-Klinik | Siegburg |
| Hamburg, Mariahilf-Klinik | Stolzenau |
| Helmstedt | Stralsund |
| Herzberg am Harz | Titisee-Neustadt |
| Hettstedt | Überlingen |
| Hildburghausen | Uelzen |
| Hildesheim | Vogelsang/Gommern |
| Hünfeld | Volkach |
| Idstein | Warburg |
| Karlsruhe | Wiesbaden |
| Kiel | Wiesbaden HSK |
| Köthen | Wipperfürth |
| Krefeld | Wittingen |
| Kronach | Wuppertal |
| Leipzig Herzzentrum | Zerbst/Anhalt |

**Supplemental Table 4: Baseline characteristics of the subgroup of cases with cardiovascular procedures**

|  | **Monthly average for 2019** | **January 2020** | **February 2020** | **March 2020** | **April 2020** | **May 2020** | **June 2020** | **July 2020** | **August 2020** | **September 2020** | **P** |
| --- | --- | --- | --- | --- | --- | --- | --- | --- | --- | --- | --- |
| Total admissions | 7427 | 8401 | 7279 | 6547 | 4933 | 6321 | 7387 | 7020 | 6389 | 2284 |  |
| **Sex** | | | | | | | | | | | |
| Male | 4712 (63%) | 5318 (63%) | 4600  (63%) | 4169 (64%) | 3248 (66%) | 4092  (65%) | 4747 (64%) | 4450 (63%) | 4128 (65%) | 1448 (63%) | n.s. |
| Female | 2715 (37%) | 3083 (37%) | 2679  (37%) | 2378 (36%) | 1685 (34%) | 2229  (35%) | 2640 (36%) | 2570 (37%) | 2261 (35%) | 836  (37%) | n.s. |
| **Age group** | | | | | | | | | | | |
| ≤ 64 years | 2596 (35%) | 2898 (34%) | 2571 (35%) | 2331 (36%) | 1681 (34%) | 2196 (35%) | 2538 (34%) | 2285 (33%) | 2142 (34%) | 750  (33%) | n.s. |
| 65-74 years | 1953 (26%) | 2211 (26%) | 1843 (25%) | 1724 (26%) | 1290 (26%) | 1689 (27%) | 1995 (27%) | 1889 (27%) | 1716 (27%) | 637  (28%) | n.s. |
| ≥ 75 years | 2878 (39%) | 3292 (39%) | 2865 (39%) | 2492 (38%) | 1962 (40%) | 2436 (39%) | 2854 (39%) | 2846 (41%) | 2531 (40%) | 897  (39%) | n.s. |
| **Charlson comorbidity index** | | | | | | | | | | | |
| 0-1 | 2854 (38%) | 3286 (39%) | 2981 (41%) | 2548 (39%) | 1708 (35%) | 2367 (37%) | 2850 (39%) | 2617 (37%) | 2395 (37%) | 984  (43%) | n.s. |
| 2-4 | 3154 (42%) | 3587 (43%) | 3012 (41%) | 2744 (42%) | 2250 (46%) | 2744 (43%) | 3185 (43%) | 3069 (44%) | 2775 (43%) | 940  (41%) | n.s. |
| ≥ 5 | 1419 (19%) | 1528 (18%) | 1286 (18%) | 1255 (19%) | 975  (20%) | 1210 (19%) | 1352 (18%) | 1334 (19%) | 1219 (19%) | 360  (16%) | n.s. |
| **Admission type^a^** | | | | | | | | | | | |
| Regular | 4514 (61%) | 5178 (62%) | 4461 (61%) | 3945 (60%) | 2545 (52%) | 3558 (56%) | 4396 (60%) | 4115 (59%) | 3752 (59%) | 1437 (63%) | n.s. |
| Urgent | 2909 (39%) | 3219 (38%) | 2816 (39%) | 2601 (40%) | 2385 (48%) | 2760 (44%) | 2988 (40%) | 2905 (41%) | 2637 (41%) | 847  (37%) | n.s. |
| **Hospital volume** | | | | | | | | | | | |
| High | 5791 (78%) | 6532 (78%) | 5648 (78%) | 5120 (78%) | 3933 (80%) | 5040 (80%) | 5816 (79%) | 5501 (78%) | 5012 (78%) | 1794 (79%) | n.s. |
| Intermediate | 1478 (20%) | 1696 (20%) | 1501 (21%) | 1310 (20%) | 958  (19%) | 1199 (19%) | 1476 (20%) | 1425 (20%) | 1308 (20%) | 454  (20%) | n.s. |
| Low | 158 (2%) | 172 (2%) | 129 (2%) | 117 (2%) | 42 (1%) | 82 (1%) | 95 (1%) | 94 (1%) | 69 (1%) | 36 (2%) | 0.024 |
| **COVID-19 case volume** | | | | | | | | | | | |
| Low | 2624 (35%) | 2939 (35%) | 2533 (35%) | 2413 (37%) | 1894 (38%) | 2350 (37%) | 2775 (38%) | 2614 (37%) | 2352 (37%) | 812  (36%) | n.s. |
| Intermediate | 3237 (44%) | 3693 (44%) | 3193 (44%) | 2860 (44%) | 2111 (43%) | 2665 (42%) | 3086 (42%) | 2916 (42%) | 2624 (41%) | 954  (42%) | n.s. |
| High | 1565 (21%) | 1769 (21%) | 1553 (21%) | 1274 (19%) | 928  (19%) | 1306 (21%) | 1526 (21%) | 1490 (21%) | 1413 (22%) | 518  (23%) | n.s. |
| ^a^ As some admissions are not classified, the total does not add to 100%. | | | | | | | | | | | |

Supplemental Figure 1: Graphical overview on case numbers of laboratory proven SARS-CoV-2 infections in Germany per day


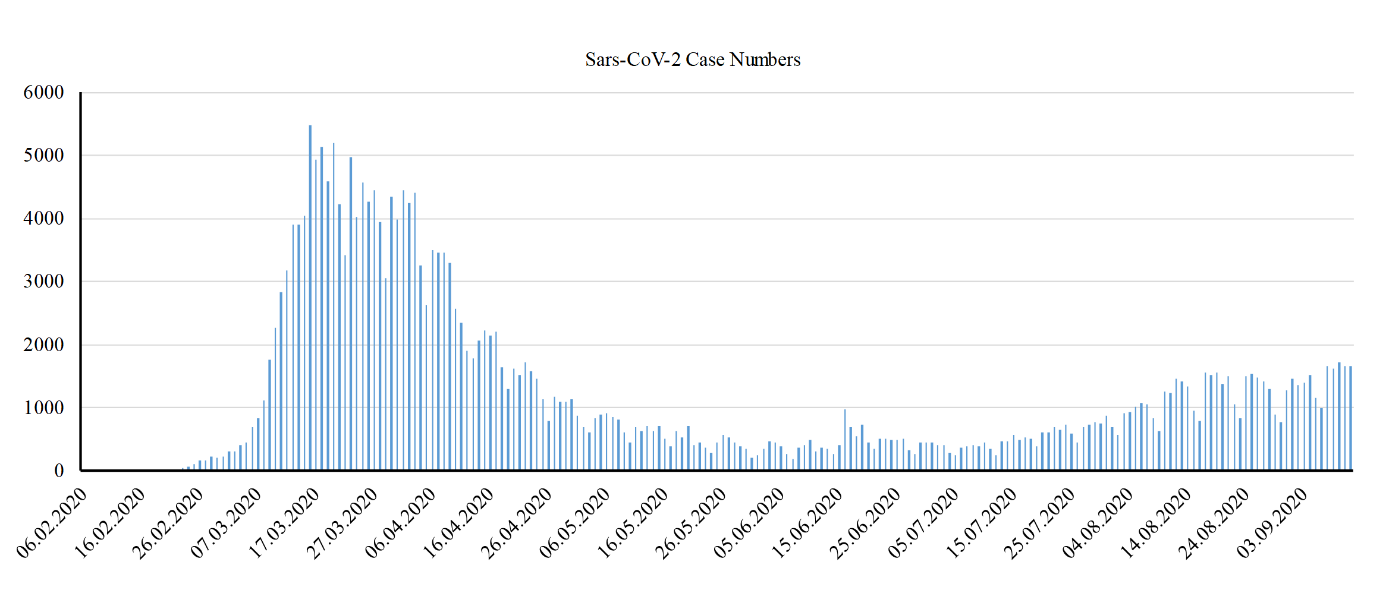

Supplement: Supplementary file 1 — Appendix S1. Supporting information. [file CLC-44-392-s001.docx]
